# A Neighborhood Analysis of the Consequences of *Quercus suber* Decline for Regeneration Dynamics in Mediterranean Forests

**DOI:** 10.1371/journal.pone.0117827

**Published:** 2015-02-23

**Authors:** Beatriz Ibáñez, Lorena Gómez-Aparicio, Peter Stoll, José M. Ávila, Ignacio M. Pérez-Ramos, Teodoro Marañón

**Affiliations:** 1 Instituto de Recursos Naturales y Agrobiología (IRNAS, CSIC), PO Box 1052, Sevilla 41080, Spain; 2 Institute for Environmental Sciences, Section Conservation Biology, University of Basel, St. Johanns-Vorstadt 10, CH-4056 Basel, Switzerland; Technion -- Israel Institute of Technology, ISRAEL

## Abstract

In forests, the vulnerable seedling stage is largely influenced by the canopy, which modifies the surrounding environment. Consequently, any alteration in the characteristics of the canopy, such as those promoted by forest dieback, might impact regeneration dynamics. Our work analyzes the interaction between canopy neighbors and seedlings in Mediterranean forests affected by the decline of their dominant species (*Quercus suber*). Our objective was to understand how the impacts of neighbor trees and shrubs on recruitment could affect future dynamics of these declining forests. Seeds of the three dominant tree species (*Quercus suber*, *Olea europaea* and *Quercus canariensis*) were sown in six sites during two consecutive years. Using a spatially-explicit, neighborhood approach we developed models that explained the observed spatial variation in seedling emergence, survival, growth and photochemical efficiency as a function of the size, identity, health, abundance and distribution of adult trees and shrubs in the neighborhood. We found strong neighborhood effects for all the performance estimators, particularly seedling emergence and survival. Tree neighbors positively affected emergence, independently of species identity or health. Alternatively, seedling survival was much lower in neighborhoods dominated by defoliated and dead *Q. suber* trees than in neighborhoods dominated by healthy trees. For the two oak species, these negative effects were consistent over the three years of the experimental seedlings. These results indicate that ongoing changes in species’ relative abundance and canopy trees’ health might alter the successional trajectories of Mediterranean oak-forests through neighbor-specific impacts on seedlings. The recruitment failure of dominant late-successional oaks in the gaps opened after *Q. suber* death would indirectly favor the establishment of other coexisting woody species, such as drought-tolerant shrubs. This could lead current forests to shift into open systems with lower tree cover. Adult canopy decline would therefore represent an additional factor threatening the recruitment of *Quercus* forests worldwide.

## Introduction

The seedling stage is one of the most vulnerable stages in the life cycle of plants as it is highly dependent on the surrounding environment [1,2,3]. The characteristics of the environment where seedlings establish can be largely influenced by plant canopy cover, which acts as a key modifier of biotic (e.g. pathogen abundance, mycorrhizal diversity, herbivory pressure) and abiotic (e.g light, temperature, soil fertility) conditions in the understory [4,5,6,7]. For example, it has been widely suggested that canopy tree species can negatively affect conspecific recruitment due to the accumulation of host-specific natural enemies, namely seed predators, pathogens and herbivores (i.e. the Janzen-Connell hypothesis; [8,9]. Moreover, seedlings of coexisting species can respond in contrasting ways to the local abiotic environment generated by a certain canopy species due to differences in their functional traits and patterns of resource acquisition and stress tolerance (i.e. the niche differentiation hypothesis, [10,11]. These non-exclusive hypotheses that tightly link the dynamics of canopy trees and understory seedlings clearly indicate that any alteration in the abundance, species composition or health status of the adult tree community might profoundly impact forest regeneration dynamics.

The death of a tree and the imminent opening of a gap is a crucial process by which canopy cover and local understory conditions are naturally changed [12,13,14]. In the last two decades, the process of gap opening in forests all around the world has been magnified due to the combined effect of several global change drivers (e.g. climate change, exotic pathogens and pests) that have caused extensive decline and mortality of several tree species worldwide (e.g. [15,16,17,18]). Selective mortality of particular tree species could drive long-term vegetation shifts due to increased seed limitation in the die-off species or due to changes in microhabitat conditions that impair its regeneration or favour the establishment of competing species [19,20]. Yet, very few studies have specifically addressed how tree decline and mortality influence regeneration dynamics with potential implications for long-term forest composition [21].

One of the main tree genera affected worldwide by die-off is *Quercus* [22,23,24]. In the Iberian Peninsula in particular, a severe tree decline affecting evergreen oak forests dominated by *Quercus ilex* and *Quercus suber* has been reported since the early 1990s [25], [22]. Underlying causes include the combination of abiotic and biotic stressors such as on-going drought linked to global warming and the attack of exotic soil-borne pathogens (mainly *Phytophthora cinnamomi*) that limit plant water uptake by destroying fine roots [26,27,18]. A fundamental characteristic of this type of drought-induced mortality is that it is not restricted to small suppressed trees, as is usually the case for background mortality in Mediterranean forests (e.g. [28,29]), but mainly affects medium to large size canopy trees. Thus, it constitutes a process with unprecedented capacity to modify patterns of seedling emergence and survival in a type of system (i.e. Mediterranean forests) inherently affected by severe regeneration problems [30,31].

In this work we aim to analyze the interaction between canopy neighbors and understory tree seedlings in Mediterranean forests affected by the decline of their dominant species (*Quercus suber*), in order to understand how neighbor-specific effects could affect the future dynamics of these declining forests. We conducted a 3-year seed sowing experiment in multiple forest sites and developed spatially- explicit neighborhood models that predicted the observed variation in tree seedling performance as a function of the size, identity, health status, abundance and distribution of adult trees and shrubs in the immediate neighborhood. Specifically, we seek to answer the following questions: (1) How does the composition of local neighborhoods affect seedling performance of coexisting tree species? We hypothesized that an increase in the abundance of defoliated and dead *Q. suber* adults in the neighborhood would translate into lower seedling performance. This prediction is based on the critical positive role that the nurse effect of adult vegetation plays for seedling regeneration in water-limited forests [30,32]. We also expected these negative effects to vary among seedling species, with shade-tolerant species being the most negatively affected by a loss of tree cover; (2) Are these neighborhoods effects consistent across years? We hypothesized the positive effects of a healthy tree cover to be particularly strong in dry years, in accordance with the Stress Gradient Hypothesis that predicts the prevalence of net positive interactions under stressful environmental conditions [33,34,35]. Moreover, we expected the magnitude of neighborhood effects to decrease as seedlings become older and develop higher stress tolerance [36,37]; and (3) What are the implications of neighborhood interactions on the successional dynamics of declining oak forests? We hypothesized that the negative effects of defoliated and dead *Q. suber* trees on establishment of dominant *Quercus* species might translate into changes in species relative abundance altering the structure and successional dynamics of these forests.

## Methods

### Ethics Statement

All necessary permits were obtained for the field studies described herein thanks to J. Manuel Fornell Fernández, Director of Los Alcornocales Natural Park.

### Study site and species

The study was conducted in the Alcornocales Natural Park (36°22’N-5°34’W), a protected area in Southwestern Spain approximately of 170 000 ha in extent. The climate is sub-humid Mediterranean, with cool, humid winters and hot, dry summers. Mean annual rainfall is 970 mm (mean 1951–1999). Mean annual temperature ranges from 14.6 to 18.4°C, with a mean monthly maximum of 36°C (July) and a mean monthly minimum of 2.8°C (January). The three study years (2010, 2011 and 2012) had contrasting weather conditions. The year 2010 was especially wet with a higher than average annual and summer rainfall (1346 mm and 40 mm, respectively), 2011 was an average year in terms of both annual and summer rainfall (1037 mm and 16 mm, respectively), whereas 2012 was an extremely dry year with 474 mm annual rainfall and 0 mm summer rainfall. The bedrock is dominated by Oligo-Miocenic sandstone that is frequently interspersed with layers of marl sediments, yielding soils rich in clay.

The Alcornocales Natural Park is situated in the Baetic-Rifean biogeographic region, which is considered a *hot spot* of biodiversity in the Mediterranean [38]. This protected area contains the largest and best conserved *Quercus suber* forests of Europe [39]. In drier and clayish lowlands, *Q. suber* forms mixed open woodlands with the evergreen, drought-tolerant *Olea europaea* var. *sylvestris*, whereas in sandier, wetter areas *Q. suber* coexists with the semi-deciduous, shade-tolerant *Quercus canariensis* Willd. (Algerian oak) forming closed forests [40]. The shrubby understory is diverse and rich in endemic taxa [41]. These forests are relatively well conserved since 1989 (declared as Natural Park), and the main management activities are the extraction of cork, game hunting and recreation [39]

### Experimental design

The field experiment was conducted in six study sites distributed in public areas across the whole Natural Park. Three of the sites were located in open woodlands of *Q. suber* and *O. europaea* (hereafter woodland sites), and the other three sites were located in closed forests of *Q. suber* and *Q. canariensis* (hereafter closed forest sites) (see S1 Fig.). The six sites covered a gradient of climate and soil conditions (see S1 Table). At each site we established a 70 x 70 m permanent plot in a topographically uniform area. Each plot was subdivided in 49 10 x 10 m subplots (6 sites x 49 subplots, 294 sampling points).

Seed sowings were conducted during the winters (January) of 2010 (first cohort) and 2011 (second cohort). Seeds of each of the three tree species were collected from at least 10 different trees throughout the Natural Park during the previous autumn and combined to make a common pool of seeds. Viable seeds were selected by flotation and stored in moist substrate at 4°C until sowed. Seeds were surface-sterilized in 10% household bleach for 5 minutes and sown at each site in two adjacent 30 x 30 cm^2^ quadrats at the center of each of the 49 subplots. Each quadrat contained three lines of seeds that were separated from each other and from the border of the quadrat by 7.5 cm. In the woodland sites, we alternatively sowed each line with three seeds of *Q. suber* or six seeds of *O. europaea*. The larger number of *Olea* seeds was chosen based on their lower germination rates [42,43]. In the closed forest sites, we alternatively sowed each line with three seeds of *Q. suber* or *Q. canariensis*. We removed the few grass species that were present at the showing points before placing the seeds. Sowing quadrats were protected with 1-cm mesh to exclude seed predators. Overall, we sowed 2646 seeds of *Q. suber*, 2646 seeds of *O. europaea* and 1323 seeds of *Q. canariensis*.

To characterize local neighborhoods, we mapped and identified all live and dead trees and shrubs around each sampling subplot. Although shrubs are frequently ignored in neighborhood studies of forest ecosystems, we decided to include them in our analysis because they constitute an important fraction of the total vegetation biomass in many Mediterranean systems and play a key role as nurse plants that increase the success of tree seedling establishment (e.g. [44,45]). We used a Leica TC 407 to map all trees with a diameter at breast height (dbh) > 2 cm located within a 15 m radius of each subplot, as well as shrubs within a 5 m radius around the subplots. We considered a radius of 5 m to be sufficiently large to detect shrub effects as most shrubs in these forests are small (height usually < 3 m) [7]. We measured the diameter at breast height of each of the trees mapped (n = 1341 trees). Due to its multi-stem growth form, shrub size was characterized by measuring the two diameters of the elliptical projection of its crown (n = 3005 shrubs). Additionally, we divided *Q. suber* trees into different categories according to health based on a standardized semi-quantitative scale widely used in the region to monitor oak decline (e.g. [46]): (1) healthy reference trees; (2) defoliated trees; and (3) dead trees. No other tree or shrub species in the study area showed symptoms of decline.

### Seedling measurements

Our measures of seedling performance were emergence, survival, height increment and photochemical efficiency. Seedling emergence was monitored in early June to ensure that most seedlings had emerged [47]. Seedlings were revisited in early October to record survival after the summer, the main period of seedling mortality in Mediterranean systems [30,47]. Survival of each emerged seedling was followed during the whole duration of the experiment (three years for the first cohort and two years for the second cohort).

To estimate seedling growth, stem height was measured in all seedlings at the end of the growth period of the three study years. The relative growth rate in height (cm) was calculated for each seedling as the fraction of height increment observed in each growing season (two growing seasons for the first seedling cohort and one for the second cohort). During the summers of 2010 and 2011, we performed in-situ chlorophyll fluorescence measurements on attached leaves using a portable, pulse-modulated fluorometer (PAM-2000, Walz Effeltrich, Germany). The photochemical efficiency of photosystem II (Fv/Fm) was measured at midday (i.e. between 12:00 and 14:00) after a 30 min dark adaptation in one 1-year old seedling per species and subplot.

### Data analyses

We used a model selection procedure [48] to estimate seedling emergence, survival, height growth and physiological condition (measured by photochemical efficiency) as a function of the characteristics of the neighborhood. Competition among seedling species was not included as we considered it to be very unlikely, given the separation between sowing points and the relatively low seedling number that emerged and survived at each sowing point. We fit separate models for each combination of forest type (woodland and closed forest), seedling cohort (2010 and 2011) and seedling species (*Q. suber* and *Q. canariensis*). Unfortunately, seedling emergence of *O. europaea* was extremely low in all sites (data not shown), which precluded us from testing neighborhood effects on this species. *Olea europaea* is naturally abundant in the seedling bank of the study sites and does not seem to suffer from relevant regeneration limitation. Therefore, we interpreted this germination failure as a problem related to low viability of the seed pool used in the experiment and/or the lack of dormancy break during the study years.

Our simplest model (*Null* model, eqn 1) estimates seedling emergence, survival, growth or photochemical efficiency using a single mean, thereby assuming that the site and the spatial distribution of woody neighbors have no impact on seedling performance. For observation *i*, this model has the form:

$$Y_{\left(i\right)}\;=\;\alpha +\varepsilon_{\left(i\right)}$$ eqn 1

where α represents the mean for the seedling performance estimator Y in each forest type, and ε is the error term. We then fit a *Site* model (eqn 2) that considered potential differences in seedling performance among the three sites of each forest type:

$$Y_{\left(i\right)}\;=\;\alpha_{Site(i)}+\varepsilon_{\left(i\right)}$$ eqn 2

Finally, the effects of neighboring trees and shrubs were incorporated into the model by adding a simple linear term:

$$Y_{\left(i\right)}\;=\;\alpha_{Site(i)}+{\beta_{Neighbor\;type(i)}*{NI}_{\left(i\right)}+\varepsilon }_{\left(i\right)}$$ eqn 3

where *NI* (neighborhood index) is the combined effect of woody neighbors on seedling performance (detailed below) and β is the slope of the effect of each neighbor type. A main motivation for this study was to compare models that make different assumptions about the nature of neighborhood interactions between canopy neighbors and tree seedlings in declining Mediterranean forests. Thus, we explicitly tested four alternate models of increasing complexity. The simplest model assumed that all neighbors had equivalent effects on the target regardless of species or health status (*All trees* model), and therefore calculated a single general β parameter. A second model differentiated between conspecific and heterospecific neighbors, and calculated separate values of β for the two classes (*Species* model). The third model separated neighbors of different species and also calculated different βs for healthy, defoliated, and dead *Q. suber* trees (*Species + Health* model). The final neighborhood model added the effect of shrubs to the best tree model, by including a separate β for shrubs (*Shrubs* model).

We tested three alternative forms of the neighborhood index (NI) where the effect of neighbors on seedling performance was: 1) exclusively a direct function of neighbor size (eqn 4); 2) a direct function of the size and an inverse function of the distance to the neighbor (eqn 5); and 3) a direct function of the size and an inverse function of the square distance to the neighbor [49] (eqn 6):

$$NI=\sum S_{i}$$ eqn 4

$$NI=\sum \frac{S_{i}}{d_{i}}$$ eqn 5

$$NI=\sum \frac{S_{i}}{d_{i}^{2}}$$ eqn 6

In these equations, *S*_*i*_ is the size of the tree or shrub *i* within the neighborhood and *d*_*i*_ its distance to the target subplot or seedling. The size of trees and shrubs was quantified differently (dbh for trees and vertical projection of the crown for shrubs), so we normalized size measurements and gave a value of 1 to the biggest tree and shrub to make parameter estimates comparable. Models were run with increasing neighborhood radii to test whether there was an optimal neighborhood radius and whether different types of neighbors influenced seedling performance at different distances [50,51,52,53]. The radii tested were from 1 to 15 m in steps of 1 m for trees, and from 1 to 5 m in steps of 1 m for shrubs.

We used the Akaike Information Criterion corrected for small sample sizes (AIC_c_) to select the best model, with lower AIC_c_ values indicating stronger empirical support for a model [54]. We allowed radii that yielded the minimum AIC_c_ to be different for each type of neighbor included in the model. Only variables that explained a significant amount of deviance (p-value < 0.05) were retained in the final best models. Seedling emergence and survival were modelled using a binomial or quasibinomial (to account for overdispersion) error distribution, and seedling growth and photochemical efficiency using a normal distribution. In growth models, where seedlings of the same subplot were considered independent, subplot was included as a random factor to control for pseudoreplication. We used pseudo R^2^ as a measure of goodness of fit in the emergence and survival models [55], and the R^2^ of observed vs. predicted values in the growth and photochemical efficiency models. All analyses were performed using R [56].

## Results

Models that included the effects of neighboring trees or shrubs on seedling performance were a better fit to the data than models that ignored them in 83% (30/36) of the combinations of forest type, seedling species, seedling cohort, year and performance estimator tested (Tables 1 and 2). Moreover, 60% of these best models (18/30) offered support for idiosyncratic effects of trees differing not only in their identity but also in their health status (*Species + Health* model). Shrubs were only included in 16% (5/30) of the best neighborhood models (*Shrubs* model, Tables 1 and 2). The majority of the best neighborhood models (27/30) included the simplest neighborhood index (NI), where neighbor effects were exclusively a function of their size (i.e. it did not consider a distance decay of neighbor effects). This indicates that the spatial configuration of neighbors was less important for seedling establishment than their overall abundance within the neighborhood. There was large variability among forest and neighbor types in the value of the optimal neighborhood radius, which ranged from 1 to 15 m (see S2 and S3 Tables). In general, optimal neighborhood radii were lower in woodland than in closed forest sites, probably due to the lower tree height in woodlands. In these sites, most neighbor effects were constrained to < 10 m, whereas in closed forests roughly half of the detected effects were >10 m. We did not detect any consistent trend regarding different optimum radius among neighbor types (S2 and S3 Tables).

**Table 1:** Comparison of alternate models for performance variables of *Quercus suber* seedlings in the woodland sites.

		AICc								
Cohort	Variable	Null	Site	All trees	Species	Species + Health	Shrubs	NI	R^2^	Explanatory variables
Cohort 1	Emergence	668.2	667.5	657.4	650.4	638.7	**628.2**	S	15.2	HET+HEA+DEF+DEA+SHR
	First-year survival	316.3	291.7	289.4	281.7	**275.5**	292.3	S	22.4	Site+HET+DEA
	Second-year survival	274.0	250.4	245.0	**236.5**	242.9	248.6	S	21.0	Site+HET+CON
	Third-year survival	255.0	**235.6**	234.8	234.2	234.1	235.8	-	11.7	Site
	First-year growth	74.5	**66.5**	66.4	67.4	67.1	66.4	-	18.4	Site
	Second-year growth	**-230.0**	-227.8	-226.4	-226.8	-225.3	-227.0	-	1.2	-
	Fv/Fm	-710.0	-716.1	**-725.2**	-726.3	-723.5	-716.2	S/D	12.6	Site+TREES
Cohort 2	Emergence	690.1	653.5	626.5	624.6	**621.0**	623.9	S	20.1	Site+HEA+DEF+DEA
	First-year survival	384.4	369.8	371.3	366.2	**336.3**	350.0	S/D	20.6	Site+HET+DEF+DEA
	Second-year survival	192.9	190.8	**185.1**	187.0	185.5	186.5	S	9.2	Site+TREES
	First-year growth	65.0	59.0	58.5	59.5	**53.8**	58.0	S/D^2^	39.0	Site+DEF+DEA
	Fv/Fm	**-330.2**	-330.1	-331.3	-329.3	-331.8	-330.0	-	1.9	-

The first two models calculate an average value of seedling performance that varies (*Site* model) or not (*Null* model) among the three woodland sites. The other four models include an additional term that accounts for the effect of neighbors on seedlings, either considering all individual trees as equivalent (*All trees* model), separating among species (*Species* model) and health status (*Species + Health* model), or adding the effect of shrubs to the best tree model (*Shrubs* model). The most parsimonious model (indicated in bold) is the one with the lowest AIC_c_. Significant explanatory variables included in the best models are detailed: TREES, all trees; CON, conspecific trees; HET, heterospecific trees; HEA, healthy *Q. suber* trees; DEF, defoliated *Q. suber* trees; DEA, dead *Q. suber* trees; SHR, shrubs. R^2^ or pseudo-R^2^ of the best model is also given. NI indicates the form of the neighborhood index included in the best model; S, size; D, distance.

**Table 2:** Comparison of alternate models for performance variables of *Quercus suber* and *Quercus canariensis* seedlings in the closed forest sites.

AIC_c_											
Species	Cohort	Variable	Null	Site	All trees	Species	Species+ Health	Shrubs	NI	R^2^	Explanatory variables
*Q. suber*	Cohort 1	Emergence	568.3	515.9	514.5	510.7	**504.1**	511.2	S	24.4	Site+HET+DEA
		First-year survival	304.6	291.6	292.1	290.0	**288.0**	292.1	S	12.7	Site+DEA
		Second-year survival	281.7	219.8	211.9	**208.1**	212.1	219.9	S	39.5	Site+HET
		Third-year survival	160.5	150.2	147.6	145.0	**140.4**	150.9	S	23.6	Site+HEA+DEA
		First-**year growth**	284.0	278.7	276.6	**273.4**	275.9	275.3	S	37.9	Site+HET
		Second-year growth	**-30.5**	-29.1	-30.4	-30.1	-28.8	-28.8	-	2.1	-
		Fv/Fm	-191.4	-203.2	**-207.3**	-206.2	-201.8	-204.6	S	29.8	Site+TREES
	Cohort 2	Emergence	453.5	429.8	422.2	418.1	**415.0**	419.0	S	18.1	Site+DEA
		First-year survival	194.5	191.2	191.8	187.4	**179.4**	181.6	S	14.7	DEA
		Second-year survival	98.7	96.6	90.6	86.9	**80.9**	98.5	S	40.0	Site+HET+HEA+DEF
		First-year growth	74.0	66.7	**61.0**	60.9	61.9	67.8	S	24.2	Site+TREES
		Fv/Fm	-328.6	-342.4	-343.1	-342.5	-351.2	**-356.9**	S	36.8	Site+HEA+DEA+SHR
*Q. canariensis*	Cohort 1	Emergence	584.5	541.3	529.1	527.3	522.6	**516.9**	S	25.2	Site+CON+DEA+SHR
		First-year survival	398.6	375.1	371.7	365.8	**353.4**	375.8	S	22.1	Site+CON+DEF+DEA
		Second-year survival	348.8	262.5	255.9	251.1	**245.7**	263.7	S	44.3	Site+HEA+DEF+DEA
		Third-year survival	185.4	183.1	181.5	178.6	**145.5**	183.2	-	32.4	Site+HEA+DEA
		First-year growth	227.7	219.6	219.3	**215.4**	215.6	220.5	S	16.8	Site+CON
		Second-year growth	-287.3	-293.2	**-303.4**	-303.5	-303.3	-294.5	S	14.7	Site+TREES
		Fv/Fm	-408.2	-418.7	-419.5	-419.8	-420.4	**-422.2**	S	20.5	Site+SHR
	Cohort 2	Emergence	380.6	366.8	363.4	362.9	**355.7**	358.3	S	20.5	Site+DEF
		First-year survival	199.4	188.3	**177.1**	181.4	186.1	187.3	S	19.0	Site+TREES
		Second-year survival	113.5	98.8	99.0	94.0	**90.9**	95.1	S	36.3	Site+HEA+DEF
		First-year growth	23.1	**16.5**	17.2	18.1	15.4	15.4	-	9.0	Site
		Fv/Fm	-176.0	-183.1	-183.2	-183.1	-182.5	**-186.1**	S	17.3	Site+SHR

The first two models calculate an average value of seedling performance that varies (*Site* model) or not (*Null* model) among the three closed forest sites. The other four models include an additional term that accounts for the effect of neighbors on seedlings, either considering all individual trees as equivalent (*All trees* model), separating among species (*Species* model) and health status (*Species + Health* model), or adding the effect of shrubs to the best tree model (*Shrubs* model). The most parsimonious model (indicated in bold) is the one with the lowest AIC_c_. Significant explanatory variables included in the best models are detailed: TREES, all trees; CON, conspecific trees; HET, heterospecific trees; HEA, healthy *Q. suber* trees; DEF, defoliated *Q. suber* trees; DEA, dead *Q. suber* trees; SHR, shrubs. R^2^ or pseudo-R^2^ of the best model are also given. NI indicates the form of the neighborhood index included in the best model; S, size; D, distance.

### Seedling emergence

**Fig 1:**
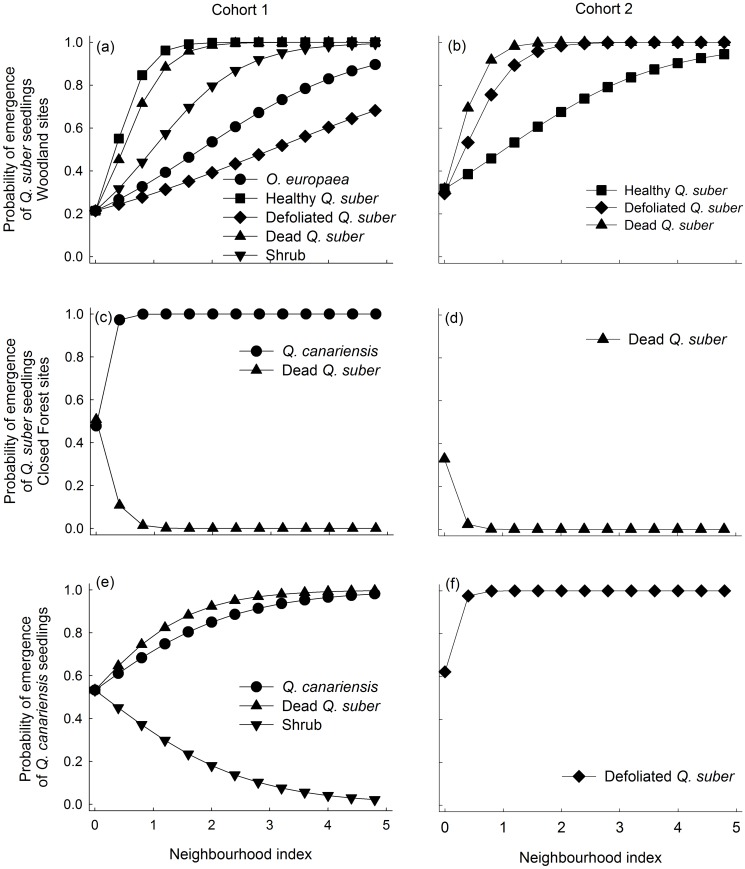
Predicted probability of emergence of *Quercus suber* seedlings in the woodland (a, b), and of *Q. suber* (c, d) and *Q. canariensis* (e, f) seedlings in the closed forest, calculated as a function of the neighborhood index (NI) using the most parsimonious models and parameters given in S4, S5 and S6 Tables. The Y-intercepts show the predicted mean emergence for the three sites in the absence of neighbors. Only neighbor types with a significant effect on emergence are shown.

Models that considered the effect of tree neighbors of different species and health status (*Species + Health* model) were the best fit for emergence of the two *Quercus* species in all combinations (6/6) of forest types and seedling cohorts. Adding shrub effects to the best tree model substantially improved model fit (i.e. decreased AIC_c_ values by > 2 units) in only 33% (2/6) of the cases (first cohort *Q. suber* emergence in woodlands and *Q. canariensis* emergence in closed forests). Positive effects on emergence were detected for all types of woody neighbors in woodland sites (i.e. positive β values; S4 Table). On the contrary, in closed forests, neighbor effects on emergence were predominantly neutral (i.e. β was not significant for most neighbor types). For both seedling species, neighbor effects on emergence were more frequently found during the extremely wet spring of 2010 than during the normal 2011 (Fig. 1).

### Seedling survival

**Fig 2:**
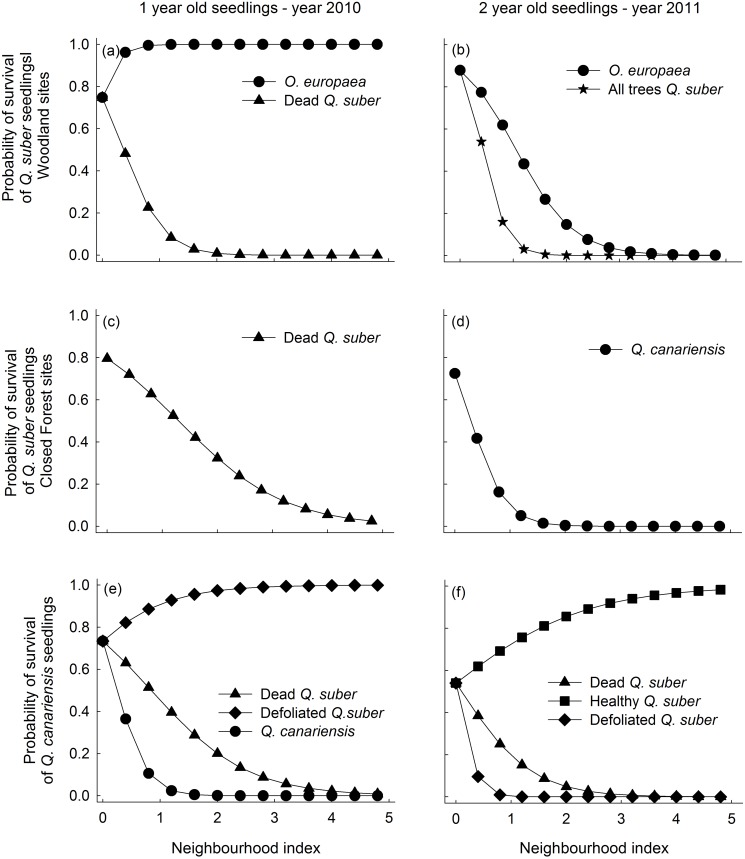
Predicted probability of survival of seedlings from the first cohort (sown in 2010) of *Quercus suber* in the woodland (a,b), and of *Q. suber* (c,d) and *Q. canariensis* (e,f) in the closed forest, calculated as a function of the neighborhood index (NI) using the most parsimonious models and parameters given in S4, S5 and S6 Tables. The Y-intercepts show the predicted mean survival for the three sites in the absence of neighbors. Only neighbor types with a significant effect on survival are shown.

**Fig 3:**
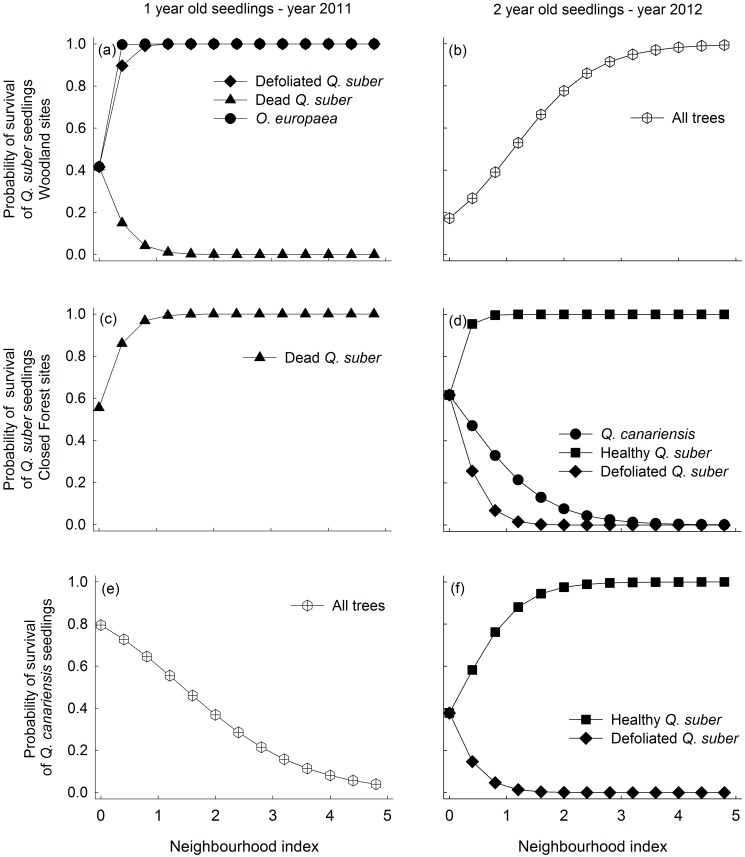
Predicted probability of survival of seedlings from the second cohort (sown in 2011) of *Quercus suber* in the woodland (a,b), and of *Q. suber* (c,d) and *Q. canariensis* (e,f) in the closed forest, calculated as a function of the neighborhood index (NI) using the most parsimonious models and parameters given in S4, S5 and S6 Tables. The Y-intercepts show the predicted mean survival for the three sites in the absence of neighbors. Only neighbor types with a significant effect on survival are shown.

The best survival models included the effect of neighbor trees in 93% (14/15) of the possible combinations of forest type, seedling species, seedling cohort and year (Tables 1 and 2). Moreover, most of these best models (10/14) separated the effect of neighbor trees of different species and health status (*Species + Health* model, Tables 1 and 2). Including shrubs never improved the fit of the best tree model (i.e. lower AIC_c_ for the *Shrub*s model in Tables 1 and 2), indicating that shrubs did not influence seedling survival during the course of the experiment. The effect of *Q. suber* trees on seedling survival varied depending on its health status, with healthy trees having predominantly neutral or positive effects and defoliated and particularly dead trees having negative impacts (Figs. 2 and 3). In woodland sites, coexistent *O. europaea* trees had a general, positive effect (i.e. positive β values, S4 Table) on 1- and 2-year old *Q. suber* seedling survival in the two cohorts. In closed forests, on the contrary, the effect of coexistent *Q. canariensis* trees on survival of the two *Quercus* species was neutral or negative (Figs. 2 and 3). Neighborhood effects were not always consistent among years. For example, tree effects on survival of 2-year old *Q. suber* seedlings in woodlands varied from strongly negative in 2011 to positive in the dry 2012 (Figs. 2b and 3b). Similar stronger positive effects in 2012 compared to 2011 were found for *Q. suber* and *Q. canariensis* seedlings of the second cohort when comparing the survival of 2-year vs. 1-year old seedlings (Fig. 3).

Three-year old seedling survival was only measured for the first cohort and in general showed fewer responses to the different neighbor types than younger seedlings. Neighborhood effects on third year survival were only detected in closed forests (Tables 1 and 2). Healthy *Q. suber* trees had strong positive effects (positive β values) and dead trees strong negative effects (negative β values) on the two seedling species, particularly on *Q. canariensis* (larger β values, see S5 and S6 Tables).

### Seedling growth

A growth model that included tree neighbor effects was the best model for 55% (5/9) of the different combinations of forest type, seedling species, seedling cohort and year tested (Tables 1 and 2). Most of these best neighborhood models pooled all trees together or differentiated only among species (*All trees* and *Species* models, Tables 1 and 2), with only one best model separating the effect of *Q. suber* trees by health status (1-year old *Q. suber* seedlings of the second cohort in woodland sites, Table 1). Including shrubs never improved the fit of the best tree model. In woodland sites, neighbor effects on growth were mainly restricted to the positive effects of dead trees (large positive β value, S4 Table). In closed forests, all trees (particularly *Q. canariensis*) had negative effects on seedling growth of the two seedling species (negative β values, see S5 and S6 Tables).

### Seedling photochemical efficiency

Models that considered the effects of tree and shrub neighbors were the best fit for seedling photochemical efficiency (Fv/Fm) in 83% (5/6) of the different combinations of forest types, seedling species and seedling cohorts tested. However, only one best model separated the effect of *Q. suber* trees by health status (*Q. suber* seedlings of the second cohort in closed forest sites, Table 2). According to this best model, healthy *Q. suber* trees had positive effects on seedling photochemical efficiency whereas dead *Q. suber* trees had negative effects (S5 Table). Neighbor shrubs had consistent positive effects on seedling photochemical efficiency during the summer for all seedling species and cohorts, but only in closed forest sites (S5 and S6 Tables).

## Discussion

Our neighborhood models provided novel evidence suggesting that species composition and health status of the tree community can largely influence the success of tree recruitment in the understory of declining Mediterranean forests. A neighborhood approach had been used before in observational studies to explore species-specific effects of neighboring trees on seedlings in tropical, cool temperate or Mediterranean forests (e.g. [57,58,37,59,60,61]). However, to our knowledge, this is the first experimental study that explores the spatiotemporal variability that complex neighborhoods (i.e. composed by trees and shrubs of different species and health status) have on consecutive plant demographic processes (seedling emergence, survival and growth) in water-limited forests affected by severe problems of decline.

### Effects of *Q. suber* decline on seedling performance

A main finding of our study is that declining *Q. suber* trees had distinctive effects than healthy trees and shrubs on the process of seedling establishment. These differences among neighbor types were not consistent across the different processes analyzed, but were much more prevalent for emergence and survival (for which the *Species + Health* model was the best one in most cases) than for growth and photochemical efficiency. Contrary to our first hypothesis, we found that all types of adult neighbors had positive effects of varying magnitude on seedling emergence. We hypothesize that these positive effects could be related to protection against waterlogging during winter and spring, as these Mediterranean forests are especially humid during those seasons. Previous studies in the study area have shown that tree seedling emergence is usually higher under the canopy than in forest gaps [62,63]. Rainfall interception by canopy trees seems to mitigate the negative effects of excess water on seedling emergence during the rainy season, particularly in forests with clayish soils of high water-holding capacity and therefore prone to suffer temporal waterlogging [64]. This could explain why the influence of neighbors on seedling emergence was particularly obvious in woodland sites, characterized by soils much richer in clay than closed forests, and where even shrubs and declining trees seem to intercept enough rainfall to have a positive effect on seedling emergence.

The effects of *Q. suber* decline were particularly relevant for the process of seedling survival. Survival of *Q. suber* and *Q. canariensis* seedlings of all ages was lower in neighborhoods dominated by defoliated and particularly dead *Q. suber* trees than in healthy neighborhoods, in accordance with our first hypothesis. In Mediterranean ecosystems, summer drought has been addressed as the most general and important cause of seedling mortality [30]. Seedling survival is frequently found to be higher under established vegetation than in open gaps due to the amelioration of extreme microclimatic conditions by woody neighbors [62,65]. Therefore, it would be possible that after the death of a tree, the loss of leaves in the crown would translate into higher light levels and evapotranspiration demand [66,67]. These changes would in turn increase abiotic stress on oak seedlings (as shown by the lower photochemical efficiency) and summer mortality rates in neighborhoods dominated by dead trees. An additional non-exclusive explanation for the lower survival in declining than in healthy neighborhoods could be related to the higher abundance of the aggressive soil-borne pathogen *Phythophthora cinnamomi* found in this type of neighborhoods [7]. Interestingly, differences in *P. cinnamomi* abundance could also explain the noticeable species-specific impacts on seedling survival found in this study, with the positive effect of *O. europaea* being related to the fact that it seems to suppress pathogens and the negative effect of *Q. canariensis* linked to its role as a pathogen reservoir [7].

Tree replacement in gaps is shaped by the species-specific ability to colonize the space opened after tree mortality [14,68]. Our two oak species differed in several relevant physiological and morphological functional traits [69] which led us to expect species-specific responses to *Q. suber* decline. In particular, we expected seedlings of the moderately shade-intolerant *Q. suber* to have an advantage over shade-tolerant seedlings of *Q. canariensis* in neighborhoods dominated by dead trees. However, we did not detect substantial support for this hypothesis, since recruitment of both species was almost null in unhealthy-tree neighborhoods, particularly in dry years (e.g. Fig. 3d,f). Therefore, in water-limited forests where positive plant-plant interactions are frequent, forests gaps that open after tree decline might not be suitable microhabitats for recruitment of dominant oak species.

### Temporal variation in neighborhood effects on recruitment

A novel aspect of our study is that it explored the consistency of neighborhood effects on tree seedling performance across several consecutive years with contrasting climatic conditions. Although we found significant neighborhood effects on seedling performance in each study year, the sign and magnitude of these effects were not consistent among years. The clearest example was the strong change in the effect of the tree canopy on second-year *Q. suber* seedling survival in woodland sites, which varied from highly negative in 2011 (an average precipitation year) to strongly positive in the extremely dry 2012 (Figs. 2b and 3b). This result supports our second hypothesis, which predicted the benefits of a nurse canopy cover to be particularly relevant under stressful environmental conditions in accordance with the Stress Gradient Hypothesis. The negative impacts on oak seedling establishment due to *Q. suber* dieback and a loss of tree cover could therefore be expected to increase in the future, as these systems become drier and warmer with ongoing climate change [70].

Temporal variation in neighborhood effects was also detected along the life of the seedlings. As predicted, 3-year old seedlings were more independent of neighborhood composition than 1- and 2-year old seedlings. A likely explanation for this pattern would be the high vulnerability and dependence of young seedlings to local environmental conditions, which rapidly decreases as they develop lignified stems and larger root systems that confer them higher stress tolerance [71,30]. It is relevant to highlight that one of the few neighbor effects found for 3-year old seedlings was a decrease in survival due to the presence of dead trees. The persistent negative effect of dead trees on seedling survival of different ages confirms the importance that tree decline could have for recruitment in these forests. Altogether, our findings imply that neighborhood studies would benefit from including a multi-year perspective in their analyses to take account of the variability shown by understory processes not only in space but also in time.

### Implication for the dynamics of forests affected by oak decline

Our modelling approach enabled us to identify how seedlings of two co-existing oak species respond to the composition of the tree and shrub neighborhood in Mediterranean forests affected by *Q. suber* decline. Our results suggest that the ongoing changes in the species relative abundance and health of these forests might alter their successional trajectory through neighbor-specific impacts on seedling performance. Theoretically, canopy gaps have been considered to provide recruitment opportunities for tree seedlings in tropical and temperate forests, allowing establishment of shade-intolerant species and increasing the diversity of tree regeneration [72,73]. However, our results offer a different picture for water-limited forests, where recruitment failure of dominant oak species in the gaps opened after *Q. suber* mortality would leave space and resources for the establishment of other coexisting woody species (e.g. drought-tolerant shrubs) with better regeneration ability and capacity to survive in open, dry microsites (e.g. drought-tolerant shrubs, [47]). A likely consequence would be the conversion of current forests into more open woodlands with lower tree cover and higher shrub cover, in a process of shrub encroachment already suggested for other water-limited forests [74,75,17].

At our study sites and in much of the Iberian Peninsula, mortality of *Q. suber* and other evergreen oaks (e.g. *Quercus ilex*) is perceived as an increasing problem but has not reached high proportions yet. However, we suggest that because plants interact at local scales, especially during regeneration, consequences of tree mortality could be larger than expected based on relative abundance of dead trees at regional scale. In fact, we found that high local densities of dead trees within 15-m neighborhoods consistently lead to strong negative effects on seedling performance in space (i.e. different forest types) and time (i.e. different years and seedling ages). The decline of the adult canopy would therefore represent an additional threat to add to the list of factors already limiting recruitment in *Quercus* forests worldwide (e.g. massive seed and seedling predation; [76,77]). Because changes in temperature, precipitation, and insect and pathogen dynamics are expected to increase the risk of forest die-off in the future [78,16], more information on post-mortality regeneration dynamics is urgently needed to predict the most likely successional trajectories and possibilities of recovery of disturbed forest ecosystems.

## Supporting Information

S1 Fig Location of the six study sites in Alcornocales Natural Park (Spain). (DOCX)

S1 Table Description of main characteristics of the six study sites located in the South (S), Center (C) and North (N) of the Alcornocales Natural Park. (DOCX)

S2 Table Optimal neighborhood radius for each neighbor type in the best models for *Quercus suber* seedlings at the woodland sites. (DOCX)

S3 Table Optimal neighborhood radius for each neighbor type in the best models for *Quercus suber* and *Quercus canariensis* seedlings at the closed forest sites. (DOCX)

S4 Table Results for the best models selected at the woodland sites for Cohorts 1 (2010) and 2 (2011) of *Quercus suber* seedlings. (DOCX)

S5 Table Results for the best models selected at the closed forest sites for Cohorts 1 (2010) and 2 (2011) of *Quercus suber* seedlings. (DOCX)

S6 Table Results for the best models selected at the closed forest sites for Cohorts 1 (2010) and 2 (2011) of *Quercus canariensis* seedlings. (DOCX)
