# Supplementary material for: A Neighborhood Analysis of the Consequences of Quercus suber Decline for Regeneration Dynamics in Mediterranean Forests
Source: PLoS One. 2015 Feb 23;10(2):e0117827. doi: 10.1371/journal.pone.0117827 (PMC4338116; doi:10.1371/journal.pone.0117827)
Supplement: S1 Fig — (DOCX) [file pone.0117827.s001.docx]

**S1 Figure** Location of the six study sites in Alcornocales Natural Park (Spain). Letters indicate North (N), Center (C) or South (S) sites in the Woodlands (WD, in blue) or Closed Forests (CF, in red).

**
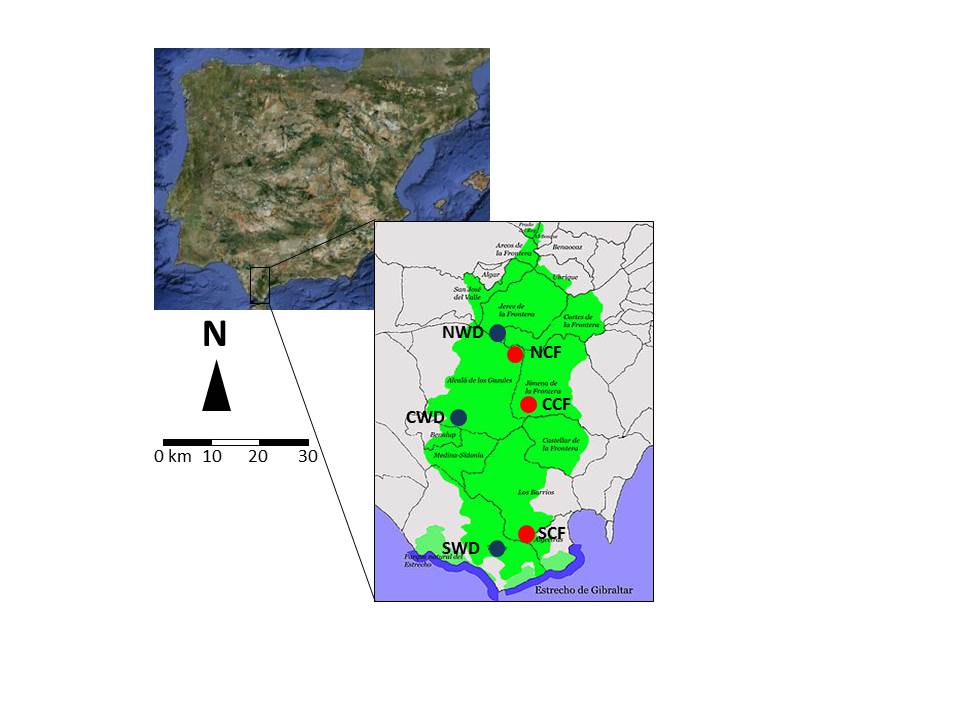
**
