# Supplementary material for: A Neighborhood Analysis of the Consequences of Quercus suber Decline for Regeneration Dynamics in Mediterranean Forests
Source: PLoS One. 2015 Feb 23;10(2):e0117827. doi: 10.1371/journal.pone.0117827 (PMC4338116; doi:10.1371/journal.pone.0117827)
Supplement: S1 Table — (DOCX) [file pone.0117827.s002.docx]

**S1 Table** Description of main characteristics of the six study sites located in the South (S), Center (C) and North (N) of the Alcornocales Natural Park. Values of texture (percentage of clay), tree basal area (m^2^/ha) and shrub crown area (m^2^/ha) represent median [P10 – P90, 10th and 90th percentiles] for the 49 sampled neighborhoods at each site. Neighborhoods are circles of 15-m (for trees) and 5-m (for shrubs) radius around each sample point. Shrub crown area is given for the most common species across the six study sites.

|  | Woodlands | | | | | |  | | | Closed forests | | | | | | |
| --- | --- | --- | --- | --- | --- | --- | --- | --- | --- | --- | --- | --- | --- | --- | --- | --- |
|  | South Site | | Center Site | North Site | |  | | | South Site | | | Center Site | | North Site | |  |
| Latitude (N) | 36º 04′ 38″ | | 36º 04′ 38″ | 36º 31′ 69″ | |  | | | 36º 06′ 09″ | | | 36º 23′ 10″ | | 36º 28′ 13″ | |  |
| Longitude (W) | 05º 33′ 05″ | | 05º 33′ 05″ | 05º 38′ 08″ | |  | | | 05º 30′ 53″ | | | 05º 31′ 52″ | | 05º 35′ 31″ | |  |
| Annual rainfall (mm) | 948.9 | | 726.4 | 973.1 | |  | | | 1067.1 | | | 1022.6 | | 1097.0 | |  |
| Mean annual T (ºC) | 16.3 | | 16.9 | 16.3 | |  | | | 15.4 | | | 17.3 | | 15.9 | |  |
| Texture | 29.62  [25.01-35.57] | 15.17  [11.76-18.21] | | 29.62  [22.99-41.97] |  | | | 12.84  [10.03-16.71] | | | 12.59  [10.01-15.59] | | 9.19  [7.72-11.73] | |  |  |
| Tree basal area |  |  | |  |  | | |  | | |  | |  | |  |  |
| *O. europaea*/*Q. canariensis* | 5.20  [2.72-7.49] | 4.18  [2.37-11.59] | | 2.57  [1.26-5.09] |  | | | 15.55  [7.93-24.01] | | | 5.45  [0-13.39] | | 14.81  [10.22-22.06] | |  |  |
| *Q. suber* _Healthy_ | 2.43  [0-5.56] | 19.02  [10.82-26.65] | | 10.26  [6.07-15.52] |  | | | 5.45  [1.50-12.48] | | | 5.61  [2.47-6.19] | | 9.56  [3.52-17.52] | |  |  |
| *Q. suber*_Defoliated_ | 5.33  [2.47-8.04] | 3.91  [1.48-7.28] | | 19.44  [12.68-28.7] |  | | | 7.18  [4.72-9.00] | | | 7.61  [2.18-10.47] | | 0.26  [0-3.52] | |  |  |
| *Q. suber* _Dead_ | 2.14  [0-7.09] | 0.00  [0-1.00] | | 0.89  [0-1.79] |  | | | 5.52  [3.22-7.64] | | | 0.00  [0-3.52] | | 0.44  [0-0.93] | |  |  |
| Shrub crown area |  |  | |  |  | | |  | | |  | |  | |  |  |
| *Pistacia lentiscus* | 198.6  [0.00-599.7] | 1276.0  [0.00-2977.0] | | 1975.0  [1442.0-3106.0] |  | | | **------** | | | 0.00  [0-0.00] | | **------** | |  |  |
| *Erica* spp. | 0.00  [0-0.00] | 0.00  [0-0.00] | | **------** |  | | | 648.1  [151.2-2472.0] | | | 3358.0  [2116.0-7810.0] | | 0.00  [0-126.4] | |  |  |
| *Phillyrea latifolia* | 0.00  [0-0.00] | 0.00  [0-0.00] | | 112.7  [0-1638.0] |  | | | 0.00  [0-0.00] | | | 0.00  [0-0.00] | | 112.7  [0-1638.0] | |  |  |
| *Crataegus monogyna* | 449.5  [150.2-714.3] | 0.00  [0-0.00] | | 1013.0  [611.2-1458.0] |  | | | **------** | | | 0.00  [0-0.00] | | 0.00  [0-0.00] | |  |  |
