# Supplementary material for: A Neighborhood Analysis of the Consequences of Quercus suber Decline for Regeneration Dynamics in Mediterranean Forests
Source: PLoS One. 2015 Feb 23;10(2):e0117827. doi: 10.1371/journal.pone.0117827 (PMC4338116; doi:10.1371/journal.pone.0117827)
Supplement: S2 Table — (DOCX) [file pone.0117827.s003.docx]

**S2 Table** Optimal neighborhood radius for each neighbor type in the best models (i.e. models with the lowest AIC_c_, Table 1) for *Quercus suber* seedlings at the woodland sites. Values within brackets indicate the neighborhood radii at which neighbor effects were detected (i.e. models with AIC_c_ lower than the null where the effect of each type of neighbor was significant, results not shown for simplicity).

| Cohort | Variable | All trees | Heterospecific | Conspecific | Healthy  *Q. suber* | Defoliated  *Q. suber* | Dead  *Q. suber* | Shrubs |
| --- | --- | --- | --- | --- | --- | --- | --- | --- |
| Cohort 1 | Emergence | - | 14[11-15] | - | 2[2-7] | 11[5-15] | 5[2-8] | 5[3-5] |
|  | First-year survival | - | 4[4-6] | - | - | - | 7[2-15] | - |
|  | Second-year survival | - | 14[9-15] | 2[2] | - | - | - | - |
|  | Third-year survival | - | - | - | - | - | - | - |
|  | First-year growth | - | - | - | - | - | - | - |
|  | Second-year growth | - | - | - | - | - | - | - |
|  | Fv/Fm | 6[3-7] | - | - | - | - | - | - |
| Cohort 2 | Emergence | - | - | - | 8[6-14] | 3[1-15] | 3[3-12] | 1[1] |
|  | First-year survival | - | 5[3-12] | - | - | 7[1-15] | 15[3-15] | - |
|  | Second-year survival | 10[10-11] | - | - | - | - | - | - |
|  | First-year growth | - | - | - | - | 7[6-15] | 4[1-15] | - |
|  | Fv/Fm | - | - | - | - | - | - | - |
