# Supplementary material for: A Neighborhood Analysis of the Consequences of Quercus suber Decline for Regeneration Dynamics in Mediterranean Forests
Source: PLoS One. 2015 Feb 23;10(2):e0117827. doi: 10.1371/journal.pone.0117827 (PMC4338116; doi:10.1371/journal.pone.0117827)
Supplement: S3 Table — (DOCX) [file pone.0117827.s004.docx]

**S3 Table** Optimal neighborhood radius for each neighbor type in the best models (i.e. models with the lowest AIC_c_, Table 2) for *Quercus suber* and *Quercus canariensis* seedlings at the closed forest sites. Values within brackets indicate the neighborhood radii at which neighbor effects were detected (i.e. models with AIC_c_ lower than the null, where the effect of each type of neighbor was significant, results not shown for simplicity).

| Species | Cohort | Variable | All trees | Heterospecifics | Conspecifics | Healthy  *Q. suber* | Defoliated  *Q. suber* | Dead  *Q. suber* | Shrubs |
| --- | --- | --- | --- | --- | --- | --- | --- | --- | --- |
| *Quercus* | Cohort 1 | Emergence | - | 1[1-15] | - | - | - | 3[1-15] | - |
| *suber* |  | First-year survival | - | - | - | - | - | 15[15] | - |
|  |  | Second-year survival | - | 4[3-7] | - | - | - | - | - |
|  |  | Third-year survival | - | - | - | 12[11-14] | - | 13[13-15] | - |
|  |  | First-year growth | - | 1[1] | - | - | - | - | - |
|  |  | Second-year growth | - | - | - | - | - | - | - |
|  |  | Fv/Fm | 1[1] | - | - | - | - | - | - |
|  | Cohort 2 | Emergence | - | - | - | - | - | 3[3] | 5[5] |
|  |  | First-year survival | - | - | - | - | - | 8[4-9] | - |
|  |  | Second-year survival | - | 11[9-13] | - | 5[4-8] | 13[4-15] | - | - |
|  |  | First-year growth | 13[12-15] | - | - | - | - | - | - |
|  |  | Fv/Fm | - | - | - | 8[5-11] | - | 14[12-15] | 3[2-4] |
| *Quercus* | Cohort 1 | Emergence | - | - | 6 [4-15] | - | - | 11[10-15] | 5[4-5] |
| *canariensis* |  | First-year survival | - | - | 2[2] | - | 10[8-11] | 15[10-15] | - |
|  |  | Second-year survival | - | - | - | 15[5-15] | 3[3-6] | 10[9-15] | - |
|  |  | Third-year survival | - | - | - | 6[4-11] | - | 13[6-15] | - |
|  |  | First-year growth | - | - | 15[9-15] | - | - | - | - |
|  |  | Second-year growth | 1[1-2] | - | - | - | - | - | - |
|  |  | Fv/Fm | - | - | - | - | - | - | 2[1-2] |
|  | Cohort 2 | Emergence | - | - | - | - | 2[1-4] | - | 5[4-5] |
|  |  | First-year survival | 15[15] | - | - | - | - | - | - |
|  |  | Second-year survival | - | - | - | 12[4-15] | 13[13-14] | - | - |
|  |  | First-year growth | - | - | - | - | - | - | - |
|  |  | Fv/Fm | - | - | - | - | - | - | 2[1-2] |
