# Supplementary material for: A Neighborhood Analysis of the Consequences of Quercus suber Decline for Regeneration Dynamics in Mediterranean Forests
Source: PLoS One. 2015 Feb 23;10(2):e0117827. doi: 10.1371/journal.pone.0117827 (PMC4338116; doi:10.1371/journal.pone.0117827)
Supplement: S4 Table — (DOCX) [file pone.0117827.s005.docx]

**S4 Table** Parameter estimates (Estimate), standard errors (SE), z-values (for emergence and survival analyses), t-values (for growth and photochemical efficiency analyses) and p-values of the partial regression coefficients, for the best models selected at the woodland sites for Cohorts 1 (2010) and 2 (2011) of *Quercus suber* seedlings. When a Site effect was found, the Intercept (α value for the South Site) and α values for the Center and North Sites are given.

| Cohort 1 (2010) | | | | | | Cohort 2 (2011) | | | | | |
| --- | --- | --- | --- | --- | --- | --- | --- | --- | --- | --- | --- |
| Variable | Parameter | Estimate | SE | z/t-value | p-value | Variable | Parameter | Estimate | SE | z/t-value | p-value |
| Emergence | Intercept | -1.30 | 0.16 | -8.26 | 0.000 | Emergence | Intercept | -0.60 | 0.10 | -5.85 | 0.000 |
|  | β_Heterospecific_ | 0.72 | 0.23 | 3.07 | 0.002 |  | α_Center_ | -0.45 | 0.16 | -2.74 | 0.006 |
|  | β_Healthy_ | 3.76 | 0.99 | 3.80 | 0.000 |  | α_North_ | -1.26 | 0.17 | -7.24 | 0.000 |
|  | β_Defoliated_ | 0.43 | 0.17 | 2.58 | 0.010 |  | β_Healthy_ | 0.75 | 0.24 | 3.18 | 0.001 |
|  | β_Dead_ | 2.77 | 0.80 | 3.49 | 0.000 |  | β_Defoliated_ | 3.04 | 0.72 | 4.22 | 0.000 |
|  | β_Shrub_ | 1.33 | 0.37 | 3.53 | 0.000 |  | β_Dead_ | 3.97 | 1.31 | 3.04 | 0.002 |
| First-year survival | Intercept | 0.96 | 0.23 | 4.22 | 0.000 | First year survival | Intercept | 0.31 | 0.21 | 1.45 | 0.147 |
|  | α_Center_ | -0.54 | 0.28 | -1.88 | 0.060 |  | α_Center_ | -1.14 | 0.26 | -4.48 | 0.000 |
|  | α_North_ | 0.93 | 0.31 | 2.99 | 0.003 |  | α_North_ | -0.80 | 0.32 | -2.48 | 0.013 |
|  | β_Heterospecific_ | 5.36 | 2.13 | 2.51 | 0.012 |  | β_Heterospecific_ | 15.60 | 4.25 | 3.67 | 0.000 |
|  | β_Dead_ | -2.90 | 0.89 | -3.25 | 0.001 |  | β_Defoliated_ | 6.24 | 1.39 | 4.48 | 0.000 |
|  |  |  |  |  |  |  | β_Dead_ | -4.06 | 1.53 | -2.66 | 0.008 |
| Second-year survival | Intercept | 1.98 | 0.34 | 5.49 | 0.000 | Second-year survival | Intercept | -0.75 | 0.38 | -1.97 | 0.049 |
|  | α_Center_ | -0.88 | 0.35 | -2.50 | 0.010 |  | α_Center_ | -1.34 | 0.42 | -3.19 | 0.001 |
|  | α_North_ | 0.88 | 0.36 | 2.41 | 0.010 |  | α_North_ | -1.13 | 0.45 | -2.51 | 0.012 |
|  | β_Heterospecific_ | -1.87 | 0.54 | -3.55 | 0.000 |  | β_All_ | 1.41 | 0.52 | 2.71 | 0.007 |
|  | β_Conspecific_ | -4.55 | 1.47 | -3.12 | 0.000 |  |  |  |  |  |  |
| Third-year survival | Intercept | 0.94 | 0.26 | 3.67 | 0.000 |  |  |  |  |  |  |
|  | α_Center_ | -1.78 | 0.42 | -4.24 | 0.000 |  |  |  |  |  |  |
|  | α_North_ | -0.17 | 0.32 | -0.54 | 0.590 |  |  |  |  |  |  |
| First-year growth | Intercept | 0.27 | 0.03 | 8.05 | 0.000 | First-year growth | Intercept | 0.16 | 0.04 | 4.17 | 0.000 |
|  | α_Center_ | -0.21 | 0.05 | -4.45 | 0.000 |  | α_Center_ | -0.04 | 0.06 | -0.76 | 0.451 |
|  | α_North_ | 0.01 | 0.04 | 0.20 | 0.840 |  | α_North_ | 0.19 | 0.07 | 2.94 | 0.004 |
|  |  |  |  |  |  |  | β_Defoliated_ | -0.67 | 0.32 | -2.10 | 0.039 |
|  |  |  |  |  |  |  | β_Dead_ | 4.05 | 1.71 | 2.36 | 0.020 |
| Second-year growth | Intercept | 0.004 | 0.01 | 0.44 | 0.000 |  |  |  |  |  |  |
| Fv/Fm | Intercept | -0.12 | 0.002 | -62.71 | 0.000 | Fv/Fm | Intercept | -0.18 | 0.01 | -26.1 | 0.000 |
|  | α_Center_ | 0.004 | 0.002 | 1.48 | 0.142 |  | α_Center_ | 0.02 | 0.01 | 1.89 | 0.062 |
|  | α_North_ | 0.005 | 0.003 | 1.99 | 0.048 |  | α_North_ | 0.001 | 0.01 | 0.38 | 0.702 |
|  | β_All_ | 0.03 | 0.009 | 3.08 | 0.002 |  |  |  |  |  |  |
