# Supplementary material for: A Neighborhood Analysis of the Consequences of Quercus suber Decline for Regeneration Dynamics in Mediterranean Forests
Source: PLoS One. 2015 Feb 23;10(2):e0117827. doi: 10.1371/journal.pone.0117827 (PMC4338116; doi:10.1371/journal.pone.0117827)
Supplement: S5 Table — (DOCX) [file pone.0117827.s006.docx]

**S5 Table** Parameter estimates (Estimate), standard errors (SE), z-values (for emergence and survival analyses), t-values (for growth and photochemical efficiency analyses) and p-values of the partial regression coefficients, for the best models selected at the closed forest sites for Cohorts 1 (2010) and 2 (2011) of *Quercus suber* seedlings. When a Site effect was found, the Intercept (α value for the South Site) and α values for the Center and North Sites are given.

|  | Cohort 1 (2010) | |  |  |  |  |  | Cohort 2 (2011) | |  |  |
| --- | --- | --- | --- | --- | --- | --- | --- | --- | --- | --- | --- |
| Variable | Parameter | Estimate | SE | z/t-value | P-value | Variable | Parameter | Estimate | SE | z/t-value | P-value |
| Emergence | Intercept | 0.21 | 0.11 | 1.96 | 0.050 | Emergence | Intercept | -0.32 | 0.12 | -2.63 | 0.009 |
|  | α_Center_ | 0.26 | 0.17 | 1.54 | 0.120 |  | α_Center_ | 0.15 | 0.25 | 0.59 | 0.554 |
|  | α_North_ | -0.84 | 0.15 | -5.53 | 0.000 |  | α_North_ | -0.80 | 0.17 | -4.84 | 0.000 |
|  | β_Heterospecific_ | 7.61 | 2.58 | 2.95 | 0.000 |  | β_Dead_ | -9.04 | 3.10 | -2.91 | 0.003 |
|  | β_Dead_ | -5.72 | 1.92 | -2.97 | 0.000 |  |  |  |  |  |  |
| First-year survival | Intercept | 1.84 | 0.32 | 5.78 | 0.000 | First year survival | Intercept | 0.22 | 0.15 | 1.43 | 0.154 |
|  | α_Center_ | -0.12 | 0.33 | -0.38 | 0.703 |  | β_Dead_ | 3.98 | 1.09 | 3.66 | 0.000 |
|  | α_North_ | -1.31 | 0.33 | -3.91 | 0.000 |  |  |  |  |  |  |
|  | β_Dead_ | -1.05 | 0.44 | -2.41 | 0.016 |  |  |  |  |  |  |
| Second-year survival | Intercept | 0.97 | 0.27 | 3.5 | 0.000 | Second year survival | Intercept | 3.76 | 1.17 | 3.20 | 0.001 |
|  | α_Center_ | 1.34 | 0.38 | 3.5 | 0.000 |  | α_Center_ | -3.07 | 0.95 | -3.23 | 0.001 |
|  | α_North_ | -1.34 | 0.33 | -4.0 | 0.000 |  | α_North_ | -3.30 | 1.03 | -3.19 | 0.001 |
|  | β_Heterospecific_ | -3.26 | 0.89 | -3.7 | 0.000 |  | β_Heterospecific_ | -1.48 | 0.68 | -2.19 | 0.029 |
| Third-year survival | Intercept | 1.51 | 0.51 | 2.97 | 0.003 |  | β_Healthy_ | 6.41 | 6.41 | 3.11 | 0.002 |
|  | α_Center_ | -1.66 | 0.47 | -3.56 | 0.000 |  | β_Defoliated_ | -3.85 | -3.86 | -2.73 | 0.006 |
|  | α_North_ | -2.91 | 0.74 | -3.91 | 0.000 |  |  |  |  |  |  |
|  | β_Healthy_ | 1.43 | 0.57 | 2.52 | 0.011 |  |  |  |  |  |  |
|  | β_Dead_ | -1.98 | 0.77 | -2.57 | 0.010 |  |  |  |  |  |  |
| First-year growth | Intercept | 0.45 | 0.04 | 9.90 | 0.000 | First year growth | Intercept | 0.96 | 0.20 | 4.82 | 0.000 |
|  | α_Center_ | -0.23 | 0.06 | -3.75 | 0.000 |  | α_Center_ | -0.54 | 0.09 | -5.65 | 0.000 |
|  | α_North_ | -0.27 | 0.09 | -3.02 | 0.000 |  | α_North_ | -0.49 | 0.11 | -4.41 | 0.000 |
|  | β_Heterospecific_ | -1.98 | 0.76 | -2.60 | 0.010 |  | β_All_ | -0.32 | 0.12 | -2.77 | 0.008 |
| Second-year growth | Intercept | 0.008 | 0.017 | 0.49 | 0.630 |  |  |  |  |  |  |
| Fv/Fm | Intercept | -0.12 | 0.01 | -19.13 | 0.000 | Fv/Fm | Intercept | -0.10 | 0.01 | -15.68 | 0.000 |
|  | α_Center_ | -0.04 | 0.01 | -4.41 | 0.000 |  | α_Center_ | -0.05 | 0.01 | -6.27 | 0.000 |
|  | α_North_ | -0.02 | 0.01 | -1.51 | 0.140 |  | α_North_ | -0.02 | 0.01 | -3.55 | 0.001 |
|  | β_All_ | -0.19 | 0.08 | -2.46 | 0.020 |  | β_Healthy_ | 0.03 | 0.01 | 3.10 | 0.003 |
|  |  |  |  |  |  |  | β_Death_ | -0.03 | 0.01 | -2.70 | 0.008 |
|  |  |  |  |  |  |  | β_Shrub_ | 0.07 | 0.03 | 2.38 | 0.020 |
