# Supplementary material for: A Neighborhood Analysis of the Consequences of Quercus suber Decline for Regeneration Dynamics in Mediterranean Forests
Source: PLoS One. 2015 Feb 23;10(2):e0117827. doi: 10.1371/journal.pone.0117827 (PMC4338116; doi:10.1371/journal.pone.0117827)
Supplement: S6 Table — (DOCX) [file pone.0117827.s007.docx]

**S6 Table** Parameter estimates (Estimate), standard errors (SE), z-values (for emergence and survival analyses), t-values (for growth and photochemical efficiency analyses) and p-values of the partial regression coefficients, for the best models selected at the closed forest sites for Cohorts 1 (2010) and 2 (2011) of *Quercus canariensis* seedlings. When a Site effect was found, the Intercept (α value for the South Site) and α values for the Center and North Sites are given.

|  | Cohort 1 (2010) | |  |  |  |  |  | Cohort 2 (2011) | |  |  |
| --- | --- | --- | --- | --- | --- | --- | --- | --- | --- | --- | --- |
| Variable | Parameter | Estimate | SE | z/t-value | P-value | Variable | Parameter | Estimate | SE | z/t-value | P-value |
| Emergence | Intercept | -0.42 | 0.12 | -3.63 | 0.000 | Emergence | Intercept | -1.44 | 0.12 | -11.96 | 0.000 |
|  | α_Center_ | 0.26 | 0.18 | 1.42 | 0.155 |  | α_Center_ | 0.56 | 0.17 | 3.23 | 0.001 |
|  | α_North_ | 1.39 | 0.21 | 6.69 | 0.000 |  | α_North_ | 0.41 | 0.30 | 1.34 | 0.177 |
|  | β_Conspecific_ | 1.03 | 0.29 | 3.52 | 0.000 |  | β_Defoliated_ | 8.00 | 2.29 | 3.50 | 0.000 |
|  | β_Dead_ | 1.18 | 0.36 | 3.29 | 0.001 |  |  |  |  |  |  |
|  | β_Shrub_ | -0.82 | 0.34 | -2.39 | 0.017 |  |  |  |  |  |  |
| First-year survival | Intercept | 0.24 | 0.16 | 1.48 | 0.137 | First year survival | Intercept | 3.98 | 0.56 | 4.51 | 0.000 |
|  | α_Center_ | 1.39 | 0.31 | 4.45 | 0.000 |  | α_Center_ | -0.78 | 0.51 | -3.45 | 0.001 |
|  | α_North_ | 0.94 | 0.27 | 3.52 | 0.000 |  | α_North_ | -2.53 | 0.71 | -2.03 | 0.042 |
|  | β_Conspecific_ | -3.93 | 1.47 | -2.68 | 0.007 |  | β_All_ | -1.95 | 0.56 | -3.51 | 0.000 |
|  | β_Defoliated_ | 1.29 | 0.46 | 2.82 | 0.005 |  |  |  |  |  |  |
|  | β_Dead_ | -1.20 | 0.40 | -3.00 | 0.003 |  |  |  |  |  |  |
| Second-year survival | Intercept | 0.00 | 0.33 | 4.09 | 0.000 | Second year survival | Intercept | 1.47 | 0.98 | 3.56 | 0.000 |
|  | α_Center_ | 1,83 | 0.38 | 8.41 | 0.000 |  | α_Center_ | -2.43 | 0.82 | -1.27 | 0.204 |
|  | α_North_ | -1.35 | 0.77 | -4.93 | 0.000 |  | α_North_ | -3.48 | 0.74 | -2.72 | 0.007 |
|  | β_Healthy_ | 0.81 | 0.29 | 2.81 | 0.005 |  | β_Healthy_ | 2.08 | 0.71 | 2.91 | 0.004 |
|  | β_Defoliated_ | -6.31 | 1.77 | -3.57 | 0.000 |  | β_Defoliated_ | -3.15 | 1.24 | -2.54 | 0.011 |
|  | β_Dead_ | -1.57 | 0.61 | -2.58 | 0.010 |  |  |  |  |  |  |
| Third-year survival | Intercept | 2.22 | 0.92 | 1.92 | 0.050 |  |  |  |  |  |  |
|  | α_Center_ | -0.02 | 0.84 | -0.56 | 0.572 |  |  |  |  |  |  |
|  | α_North_ | -1.77 | 0.81 | 0.55 | 0.581 |  |  |  |  |  |  |
|  | β_Healthy_ | 3.50 | 0.97 | 3.59 | 0.000 |  |  |  |  |  |  |
|  | β_Dead_ | -3.93 | 1.03 | -3.83 | 0.000 |  |  |  |  |  |  |
| First-year growth | Intercept | 0.15 | 0.06 | 3.34 | 0.001 | First year growth | Intercept | 0.33 | 0.06 | 5.85 | 0.000 |
|  | α_Center_ | -0.09 | 0.05 | -5.68 | 0.000 |  | α_Center_ | -0.34 | 0.07 | -5.00 | 0.000 |
|  | α_North_ | 0.35 | 0.06 | 5.67 | 0.000 |  | α_North_ | -0.14 | 0.11 | -1.34 | 0.187 |
|  | β_Conspecific_ | -0.11 | 0.04 | -2.50 | 0.014 |  |  |  |  |  |  |
| Second-year old growth | Intercept | 0.10 | 0.01 | 7.26 | 0.000 |  |  |  |  |  |  |
|  | α_Center_ | -0.08 | 0.02 | -4.75 | 0.000 |  |  |  |  |  |  |
|  | α_North_ | 0.03 | 0.06 | 0.52 | 0.605 |  |  |  |  |  |  |
|  | β_All_ | -0.48 | 0.13 | -3.69 | 0.000 |  |  |  |  |  |  |
| Fv/Fm | Intercept | -0.11 | 0.003 | -44.25 | 0.000 | Fv/Fm | Intercept | -0.16 | 0.01 | -13.50 | 0.000 |
|  | α_Center_ | -0.02 | 0.005 | -4.50 | 0.000 |  | α_Center_ | -0.07 | 0.02 | -3.97 | 0.000 |
|  | α_North_ | 0.00 | 0.004 | -0.50 | 0.594 |  | α_North_ | -0.03 | 0.02 | -1.85 | 0.070 |
|  | β_Shrub_ | 0.25 | 0.109 | 2.32 | 0.024 |  | β_Shrub_ | 0.17 | 0.08 | 2.21 | 0.031 |
